# Glucose triggers stomatal closure mediated by basal signaling through HXK1 and PYR/RCAR receptors in Arabidopsis

**DOI:** 10.1093/jxb/ery024

**Published:** 2018-02-10

**Authors:** Yan Li, Shanshan Xu, Zhiwei Wang, Lingchao He, Kang Xu, Genxuan Wang

**Affiliations:** 1 Institute of Ecology, College of Life Sciences, Zhejiang University, Hangzhou, China; 2 Natural History Research Center, Shanghai Natural History Museum, Branch of Shanghai Science & Technology Museum, Shanghai, China

**Keywords:** ABA signaling, Ca^2+^, glucose, hexokinase1, NO production, ROS production, stomatal closure

## Abstract

Sugars play important roles in regulating plant growth, development, and stomatal movement. Here, we found that glucose triggered stomatal closure in a dose- and time-dependent manner in Arabidopsis. Pharmacological data showed that glucose-induced stomatal closure was greatly inhibited by catalase [CAT; a reactive oxygen species (ROS) scavenger], diphenyleneiodonium chloride (DPI; an NADPH oxidase inhibitor), lanthanum chloride (LaCl_3_; a Ca^2+^ channel blocker), EGTA (a Ca^2+^ chelator), and two nitrate reductase (NR) inhibitors, tungstate and sodium azide (NaN_3_), while it was not affected by salicylhydroxamic acid (SHAM; a peroxidase inhibitor). Moreover, glucose induced ROS and nitric oxide (NO) production in guard cells of Arabidopsis. The ROS production was almost completely removed by CAT, strongly restricted by DPI, and was not affected by SHAM. NO production was partially suppressed by tungstate and NaN_3_, and the levels of NO were significantly reduced in the *nia1-1nia2-5* mutant. Additionally, glucose-triggered stomatal closure was significantly impaired in *gin1-1*, *gin2-1*, *pyr1pyl1pyl2pyl4*, *abi1-1*, *ost1*, *slac1-4*, *cpk6-1*, and *nia1-1nia2-5* mutants. Likewise, the reductions in leaf stomatal conductance (*g*_s_) and transpiration rate (E) caused by glucose were reversed in the above mutants. These results suggest that glucose-triggered stomatal closure may be dependent on basal signaling through PYR/RCAR receptors and hexokinase1 (HXK1).

## Teaser

Glucose-triggered stomatal closure is dependent on basal ABA signaling through PYR/RCAR receptors, CDPK6, and glucose signaling mediated by hexokinase1 (HXK1) in Arabidopsis.

## Introduction

Stomata are composed of a pair of guard cells in the aerial parts of the plants and play vital roles in controlling both the intake of CO_2_ for photosynthesis and transpirational water loss from the plant (Hetherington and Woodward, 2003). The stomatal pore apertures can be modulated by multiple environmental cues such as CO_2_ concentration, light intensity, air humidity, drought stress, as well the plant hormone abscisic acid (ABA) (Fan *et al.*, 2004; Acharya and Assmann, 2009). ABA is synthesized from a carotenoid precursor, which is critical in regulating plant development and stress responses (Finkelstein *et al.*, 2002; Nambara and Marion-Poll, 2005; Israelsson *et al.*, 2006; Yoshida *et al.*, 2006). Recent studies have revealed the functional and structural mechanisms underlying ABA perception and its downstream signaling network (Fujii *et al.*, 2009; Ma *et al.*, 2009; Park *et al.*, 2009). The central signaling module of the ABA pathway consists of three major components: the ABA receptors pyrabactin resistance1/PYR1-like/regulatory component of ABA receptor (PYR1/PYL/RCAR), type 2C protein phosphatases (PP2Cs), and subclass2 Snf1-related kinases (SnRK2s) (Ma *et al.*, 2009; Park *et al.*, 2009). In the absence of ABA, PP2Cs inactivate SnRK2s by dephosphorylating a key serine residue in the activation loop and developing physical complexes with SnRK2s, thereby preventing the entry of the substrates (Ma *et al.*, 2009; Park *et al.*, 2009). Binding of ABA to the intracellular PYR/RCAR receptors triggers a conformational change, which permits them to combine with and inactivate PP2Cs. The inactivity of PP2Cs releases its inhibitory effects on SnRK2s, and the activated SnRK2s further phosphorylate target components of their downstream signaling pathways (Ma *et al.*, 2009; Park *et al.*, 2009). It is well known that ABA is a key endogenous factor mediating stomatal closure in response to various environmental stresses (Nambara and Marion-Poll, 2005; Acharya and Assmann, 2009; Bari and Jones, 2009). Under stress, the accumulated endogenous ABA combines with the ABA receptors PYR1/PYL/RCAR, which induces the interactions with a group of PP2Cs, ABI1 and ABI2, and thus relieves the inhibition of PP2Cs on open stomata 1 (OST1) (Ma *et al.*, 2009; Park *et al.*, 2009). Subsequently, OST1 can directly activate slow type anion channel 1 (SLAC1) responsible for anion efflux via protein phosphorylation, finally inducing stomatal closure (Fujii *et al.*, 2009; Geiger *et al.*, 2009; Park *et al.*, 2009). Additionally, OST1 kinases can phosphorylate the plasma membrane NADPH oxidase, which is the most studied reactive oxygen species (ROS)-producing enzyme in mediating stomatal closure (Sirichandra *et al.*, 2009). ROS production by NADPH oxidase has been proved to be involved in ABA-, methyl jasmonate (MeJA)-, ethylene-, ozone-, darkness-, and *Chlorella*-induced stomatal closure (Pei *et al.*, 2000; Kwak *et al.*, 2003; Desikan *et al.*, 2004; Suhita *et al.*, 2004; Joo *et al.*, 2005; Bright *et al.*, 2006; Munemasa *et al.*, 2007; Li *et al.*, 2014). In addition to ROS production, nitric oxide (NO) production, cytosolic free calcium concentration ([Ca^2+^]_cyt_), Ca^2+^-permeable channels, and Ca^2+^-dependent protein kinases (CDPKs) were also involved in the ABA-induced stomatal closure (Allen *et al.*, 2000; Mori *et al.*, 2006).

Glucose can function like a hormone and has emerged as a key signaling molecule that modulates many vital physiological processes in photosynthetic plants. Previous studies have shown that there are complicated relationships between glucose and ABA signaling pathways (Rolland *et al.*, 2006; Zhang *et al.*, 2008). For example, glucose can control the expressions of genes in ABA biosynthesis and signaling events during seedling development (Rolland *et al.*, 2002, 2006). For example, Glucose insensitive 1 (GIN1) encodes cytosolic short-chain dehydrogenase/reductase, which is responsible for converting xanthoxin to ABA-aldehyde in ABA biosynthesis (W.-H. Cheng *et al.*, 2002). Phenotype analysis further showed that the *gin1-1* mutant was insensitive to glucose and had an increased rate of water loss, leading to symptoms of wilting and withering under low relative humidity and water stress (W.-H. Cheng *et al.*, 2002). Additionally, genetic analysis revealed that GIN1 acted downstream of the glucose sensor hexokinases (HXKs) and gin1 was epistatic to HXKs in the glucose signaling pathway (Zhou *et al.*, 1998). HXKs are the enzymes catalyzing the phosphorylation of hexose sugars in the first step of the glycolytic pathway. Previous studies have revealed that Glucose insensitive 2 (GIN2) encodes a HXK1 in the plant glucose signaling network and acts as a glucose sensor to co-ordinate light, nutrient, and hormone signaling networks in regulating plant growth and development in response to environmental changes (Moore *et al.*, 2003). The loss-of-function HXK1 mutant *gin2-1* was isolated using a two-step genetic screen in Arabidopsis. The *gin2-1* mutant contains a nonsense mutation and has reduced HXK1 transcripts and truncated HXK1 protein accumulation, leading to decreased enzymatic catalytic activity (Moore *et al.*, 2003). The mutant is specifically insensitive to glucose but sensitive to osmotic changes. Notably, compared with the wild type, the *gin2-1* mutant had a higher stomatal conductance (*g*_s_) and transpiration rate (E); however, plants overexpressing AtHKT1 in guard cells had a reduced *g*_s_ and E in Arabidopsis (Kelly *et al.*, 2013). A similar phenomenon was observed in citrus plants (Kelly *et al.*, 2013; Lugassi *et al.*, 2015). Kelly *et al.* (2013) also found that sucrose triggered guard cell-speciﬁc NO production via HXK and ABA in tomato. These results revealed that HXK1 mediated stomatal closure in Arabidopsis, tomato, and citrus plants (Kelly *et al.*, 2013; Lugassi *et al.*, 2015). Notably, previous work has indicated that trehalase, which speciﬁcally hydrolyzed trehalose into glucose, up-regulates stomatal closure in Arabidopsis (Van Houtte *et al.*, 2013). Our published data further showed that glucose and mannose could induce stomatal closure mediated by ROS production mainly via NADPH oxidases, Ca^2+^, and the water channel in *Vicia faba* (Li *et al.*, 2016). A new study has also revealed that G-protein signaling protein is involved in d-glucose-triggered stomatal closure (Hei *et al.*, 2017). However, it remains largely unknown whether GIN1, GIN2, PYR/RCAR, OST1, [Ca^2+^]_cyt_, the Ca^2+^ channel, CDPK6, nitrate reductase (NR), and SLAC1 are required in glucose-triggered stomatal closure in Arabidopsis.

Unlike *V. faba* and tomato, working with certain dicotyledons (e.g. *Arabidopsis thaliana*) enables us to use existing mutants to explore the mechanism underlying glucose-triggered stomatal closure. Due to species specificity, differences in stomatal anatomy and responses exist among Arabidopsis, *V. faba*, and tomato. Therefore, we further tested whether glucose can trigger stomatal closure in Arabidopsis and, if so, (i) whether glucose-induced stomatal closure is dependent on ROS and NO production, the [Ca^2+^]_cyt_, and the Ca^2+^ channel in Arabidopsis; and (ii) whether GIN1, GIN2, PYR/RCAR, OST1, CDPK6, NR, and SLAC1 are involved in glucose-triggered stomatal closure.

## Materials and methods

### Plant material and growth conditions

The *A. thaliana* seeds were surface sterilized in 70% ethanol for 10 min, and then sown in Petri dishes (1 × 0.15 cm) containing half-strength Murashige and Skoog (MS) solid media with 0.8% (w/v) agar and 1.5% (w/v) sucrose. The seeds were vernalized at 4 °C in the dark for 2 d and transferred into pots (6 cm×8 cm) containing a mixture of growing medium:vermiculite (3:1, v/v) after a 7 d germination. The plotted plants were put in artificial intelligence-controlled chambers with a temperature of 23 °C day/21 °C night, relative humidity of 70%, photosynthetic active radiation (PAR) of 100 μmol m^–2^ s^–1^, and a photoperiod of 12 h light/12 h dark, and were watered daily. Four weeks later, fully expanded leaves in the plants were selected and used for further experiments.

Ecotypes Columbia (Col), Landsberg erecta (Ler), and Wassilewskija (Ws), and mutant lines provided model materials for this study. The mutants *ost1/snrk2.6* (Salk_0677550C in the Col-0 accession), *abi1-1* (Salk_076309C in the Col-0 accession), *slac1-4* (Salk_137265 in the Col-0 accession), *gin1-1* (CS6146 in the Ws accession), *gin2-1* (CS6383 in the Ler accession), and *nia1-1nia2-5* (CS2356 in the Col-0 accession) were obtained from the Arabidopsis Biological Resource Center (ABRC). The *pyr1pyl1pyl2pyl4* mutant (1124C in the Col-0 accession) was gifted by Sean R. Cutler from the University of California, Riverside. The *cdpk6* mutant (*cpk6-1* in the Col-0 accession) was kindly provided by Julian I. Schroeder from University of California, San Diego. All the mutants mentioned above have been genetically identified.

### Chemicals

The molecular probe 2',7'-dichlorofluorescein diacetate (H_2_DCF- DA; Sigma-Aldrich, St Louis, MO, USA) and the NO fluorescent probe 4-amino-5-methylamino-2',7'-difluorofluorescein diacetate (DAF-FM DA; Invitrogen, Eugene, OR, USA) were dissolved in DMSO to produce a stock solution, which was aliquoted. Catalase (CAT, bovine liver), d-glucose, diphenyleneiodonium chloride (DPI), MES, EGTA, lanthanum chloride (LaCl_3_), sodium azide (NaN_3_), salicylhydroxamic acid (SHAM), and tungstate were obtained from Sigma-Aldrich. The remaining chemicals of the highest analytical grade were bought from Chinese companies.

### Stomatal bioassay

Stomatal bioassay experiments were performed as described (Li *et al.*, 2014, 2016) with slight modification. Briefly, the epidermis was first peeled off carefully from the abaxial surface of the youngest, fully expanded leaves of 4-week-old plants, and then cut into strips. The strips of epidermis were incubated in opening buffer (10 mM MES, 50 mM KCl, pH 6.15) for 2 h under 22–25 °C and a photon flux density of 100 μmol m^–2^ s^–1^ for stomatal opening. Once the stomata were fully open, the epidermal strips were treated with glucose solutions of different concentrations (0, 1, 10, 25, 50, 100, 150, and 200 mM) for another 2 h. In the treatment groups, where inhibitors or scavengers (DPI, SHAM, CAT, EGTA, LaCl_3_, NaN_3_, and tungstate) were needed (Potikha *et al.*, 1999; Pei *et al.*, 2000; Yamasaki and Sakihama, 2000; Zhang *et al.*, 2001; Bright *et al.*, 2006; He *et al.*, 2013), they were added 30 min prior to the glucose treatments. To elucidate the effects of glucose on stomatal movement at the leaf level, the fully expanded leaves of Arabidopsis were kept under light (100 μmol m^–2^ s^–1^) for 3 h and then treated with sterile water and glucose solution (100 mM) for another 2 h. Then, the epidermal strips were excised and observed immediately by microscopy. Stomata were digitized with a Canon PowerShot G10 camera coupled to a DSZ5000X microscope (UOP, Chongqing, China). The width and length of stomatal pores were assessed using the Image-Pro plus6.0 software (Media Cybernetics, Silver Springs, MD, USA). The stomatal aperture was calculated as pore width/length (Allen *et al.*, 2000). The experiments were repeated three times. A total of 50 stomata were selected as samples in each experiment. To obtain the time response course, the stomatal aperture was examined at 30 min intervals. Sixty stomata from three repetive experiments were measured in total.

### Leaf stomatal conductance and transpiration rate measurements

When the Arabidopsis plants were 4 weeks old, we sprayed 100 mM glucose solution and sterile water with hand sprayers onto the fully expanded leaves which had acclimated to light for 3 h in both the treatment and control groups, making both sides of the leaves uniformly wet. After a 2 h treatment, six fully expanded leaves were randomly selected in separate plants in different treatment groups and the *g*_s_ and E were measured with a portable photosynthesis system (Li-6400; Li-Cor Inc., Lincoln, NE, USA). During the experiment, the assimilation chamber was set to an air temperature of 25 °C, 500 ml min^−1^ air flow, and 380 ppm ambient CO_2_ concentration. Data were recorded after 3–4 min, when photosynthesis reached the steady state. When Arabidopsis leaves were small and do not fit on the 2 × 3 cm leaf chamber, we photographed the leaves using a Canon PowerShot G10 camera and calculated the leaf areas with Image-Pro plus6.0 software (Media Cybernetics, Silver Springs, MD, USA). The raw data obtained by the gas exchange system were converted to flux rates per unit of processed leaf area (Vahisalu *et al.*, 2008).

### ROS and NO measurements in guard cells

After the treatment in the stomatal bioassay experiments described above, the epidermal strips were loaded with 50 μM H_2_DCF-DA or 10 μM DAF-FM DA in the dark at room temperature. After 30 min, the excess dye was washed three times with opening buffer. Fluorescence photographs of guard cells were taken using a Canon PowerShot G10 camera coupled to a DSZ5000X microscope (UOP, Chongqing, China) and a confocal laser scanning microscope (excitation 490 nm; emission 515 nm) (LSM 710; Zeiss, Jena, Germany). Acquired fluorescence images were analyzed with Image-Pro plus6.0 software (Media Cybernetics). Average fluorescence intensities of treated groups were normalized to the value of the control groups, which was taken as 100% (Desikan *et al.*, 2006; Khokon *et al.*, 2010*a*). The experiment was repeated three times and the epidermal strips were selected from three individual leaves on separate plants for each replicate in every treatment group.

### Statistical analysis

Statistical analyses were performed in SPSS13 [analysis of covariance (ANCOVA) SPSS13, SPSS Inc., Chicago IL, USA]. Values of stomatal aperture, *g*_s_, E, ROS, and NO production were compared through the ANOVA procedure individually. Significance among treatments were based on *P*-values determined by the least significant difference (LSD) test (*P*<0.05).

## Results

### Glucose-induced stomatal closure in epidermal strips of Arabidopsis

**Fig. 1.**
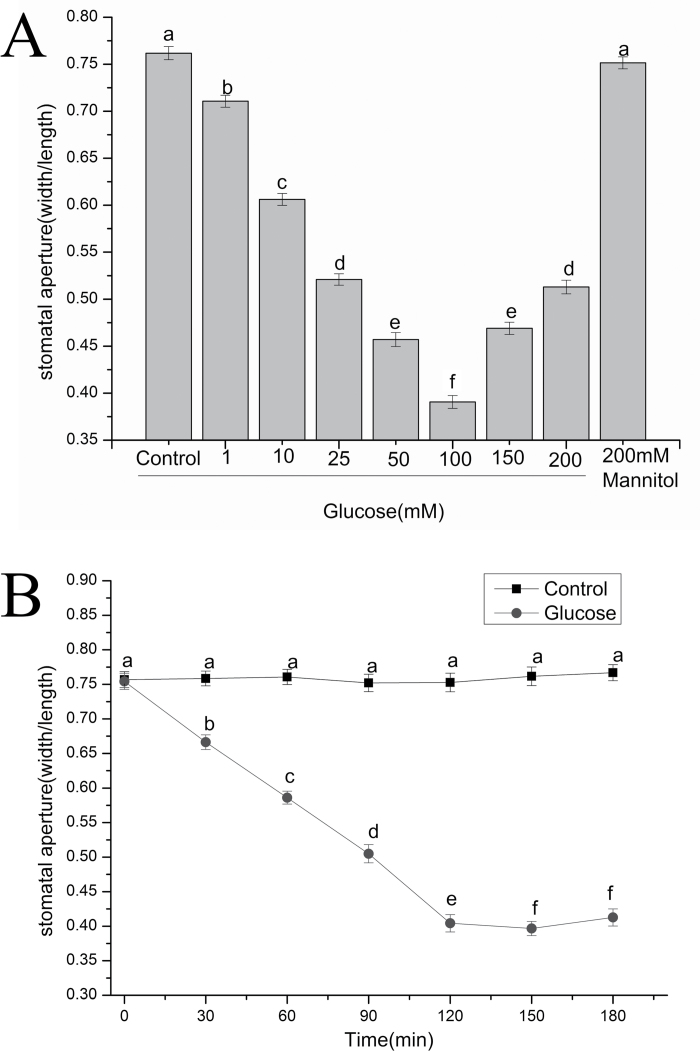
Glucose induced stomatal closure in Arabidopsis. (A) The dosage effect of glucose-induced stomatal closure. Abaxial epidermal strips of Arabidopsis were pre-incubated in MES–KCl buffer for 2 h under light, and then treated with different concentrations of glucose. The stomatal apertures were then measured. Each bar represents the mean ±SE from three independent experiments (*n*=150). (B) Time course analysis of stomatal closure triggered by 100 mM glucose solution, and stomatal apertures were examined every 30 min. Values are the means of 60 measurements ±SE of three biological repeats. Filled square, control; filled circle, glucose. Means with different letters denote statistically significant differences among different treatments as determined by ANOVA (LSD test, *P*<0.05).

Our recent studies have revealed that glucose can induce stomatal closure in a dose- and time-dependent manner in *V. faba* (Li *et al.*, 2016). To determine the effect of glucose on Arabidopsis stomatal movement, the abaxial epidermal peels of wild-type Arabidopsis (Col-0) were treated with mannitol and differing concentrations of glucose solution for 2 h under light. After the treatment with 1, 10, 25, 50, 100, 150, and 200 mM glucose solution and 200 mM mannitol (serving as an osmotic control) for 2 h, stomatal apertures were reduced by 5.1% (*P*<0.001), 15.6% (*P*<0.001), 24.1% (*P*<0.001), 30.5% (*P*<0.001), 37.1% (*P*<0.001), 29.3% (*P*<0.001), 24.9% (*P*<0.001), and 1.0% (*P*=0.2818) compared with the control, respectively (Fig. 1A), showing that glucose caused stomatal closure in a dose-dependent manner. The threshold appeared in 100 mM glucose, while the effects at higher concentrations were less significant (Fig. 1A). There was also no significant change in stomatal aperture in the 200 mM mannitol group (Fig. 1A). In addition, Fig. 1B indicated that 100 mM glucose triggered stomatal closure in a time-dependent manner. The maximum effect appeared 2 h later after treatment, and stomatal apertures decreased by 35.0% (*P*<0.001) compared with the control treatment.

### Glucose-induced stomatal closure is mediated by GIN2 and GIN1

**Fig. 2.**
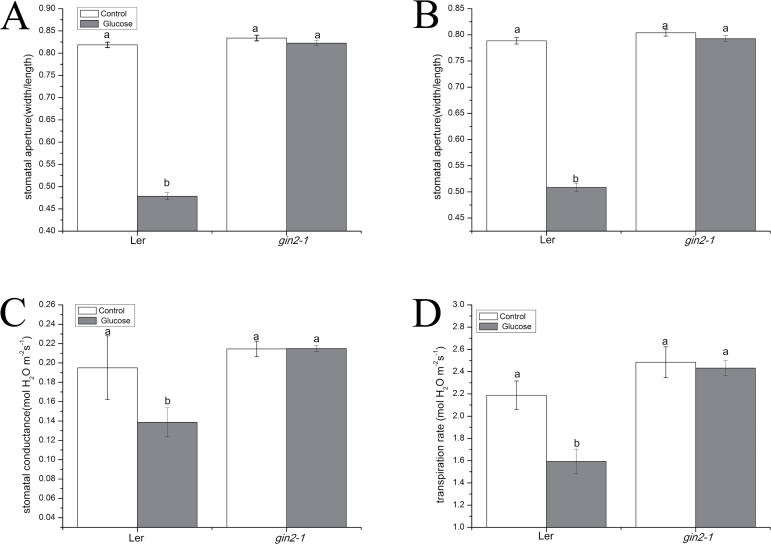
Glucose-induced stomatal closure was dependent on hexokinase1 in Arabidopsis. (A) Effects of glucose (100 mM) on stomatal apertures in epidermal strips of wild-type Arabidopsis (Ler) and glucose-insensitive mutants (*gin2-1*). These data indicate the means ±SE of three biological repeats from three independent experiments (*n*=150 per bar). (B) Effects of glucose on stomatal apertures in intact leaves of wild-type Arabidopsis (Ler) and glucose-insensitive mutants (*gin2-1*). Each bar demonstrates the means ±SE of three biological repeats (*n*=150). (C and D) Effects of sterile water and 100 mM glucose solution on leaf *g*_s_ and E of wild-type Arabidopsis (Ler) and glucose signaling mutants (*gin2-1*). Each bar represents the means ±SE (*n*=6). Different letters above the bars demonstrate statistically significant differences among treatments (LSD test, *P*<0.05). White bar, control; gray bar, glucose.

**Fig. 3.**
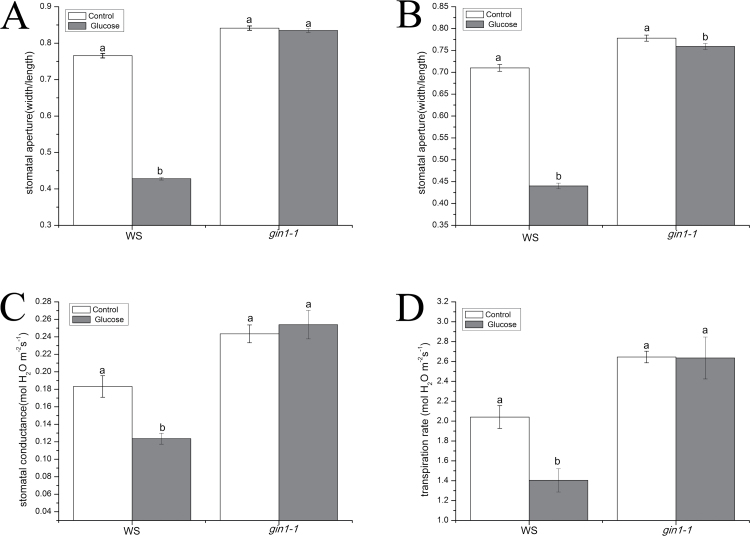
Glucose signaling mediated glucose-induced stomatal closure in Arabidopsis. (A) Effects of glucose (100 mM) on stomatal apertures in epidemal strips of wild-type Arabidopsis (Ws) and glucose-insensitive mutants (*gin1-1*). These data indicate the means ±SE of three biological repeats (*n*=150 per bar). (B) Effects of glucose (100 mM) on stomatal apertures in intact leaves of wild-type Arabidopsis (Ws) and glucose-insensitive mutants (*gin1-1*). Each bar shows the means ±SE from three independent experiments (*n*=150). (C and D) Changes of leaf *g*_s_ and E of Arabidopsis (Ws) and glucose-insensitive mutants (*gin1-1*) in response to sterile water and 100 mM glucose. Each bar represents the mean ±SE (*n*=6). Different letters above the bars denote statistically significant differences among treatments (LSD test, *P*<0.05). White bar, control; gray bar, glucose.

To clarify the role of sugar sensing in glucose-induced stomatal closure, we tested the effect of glucose on stomatal aperture in two Arabidopsis HXK-dependent glucose signaling mutants *gin2-1* (in Ler ecotype) and *gin1-1* (in Ws ecotype). As was demonstrated in Figs 2 and 3, the stomatal apertures decreased by 41.5% (*P*<0.001), 44.1% (*P*<0.001), 1.4% (*P*=0.165), and 0.8% (*P*=0.440) in epidermal peels of Ler, Ws, *gin2-1*, and *gin1-1* plants, respectively, at 2 h after treatments with 100 mM glucose solution, in comparison with the control treatment. These result showed that glucose induced stomatal closure in Ler and Ws, while *gin2-1* and *gin1-1* mutants had no obvious decrease in stomatal apertures in response to glucose treatment. The treatment with 100 mM glucose led to 35.5% (*P*<0.001), 38.0% (*P*<0.001), 1.4% (*P*=0.164), and 2.7% (*P*=0.033) reductions in stomatal apertures in intact leaves of Ler, Ws, *gin2-1*, and *gin1-1* after 2 h, compared with the control (Figs 2, 3). Briefly, after a 2 h treatment with 100 mM glucose solution, *gin2-1* and *gin1-1* had greater stomatal apertures in epidermal peels of intact leaves compared with Ler and Ws. To address further the role of HXK-dependent glucose signaling in the regulation of the stomatal response, we tested leaf *g*_s_ and E under steady-state conditions in intact Arabidopsis rosettes of Ler, Ws, *gin2-1*, and *gin1-1.* In the control treatment, leaf *g*_s_ of Ler, Ws, *gin2-1*, and *gin1-1* was reduced by 28.9% (*P*<0.001), 32.6% (*P*<0.001), –0.2% (*P*=0.968), and –4.3% (*P*=0.533), and leaf E was decreased by 27.2% (*P*<0.001), 31.2% (*P*<0.001), 2.1% (*P*=0.750), and 0.4% (*P*=0.960) correspondingly after treatment with 100 mM glucose solution for 2 h (Figs 2, 3). These results indicated that the glucose-induced reductions in stomatal apertures, *g*_s_, and E in the wild type were almost completely restored in *gin2-1* and *gin1-1* mutants, showing that GIN2 and GIN1 may play a fundamental role in regulating stomatal responses to glucose in Arabidopsis.

### Glucose-induced stomatal closure is modulated by functional PYR/RCAR, ABI1, OST1, and SLAC1

**Fig. 4.**
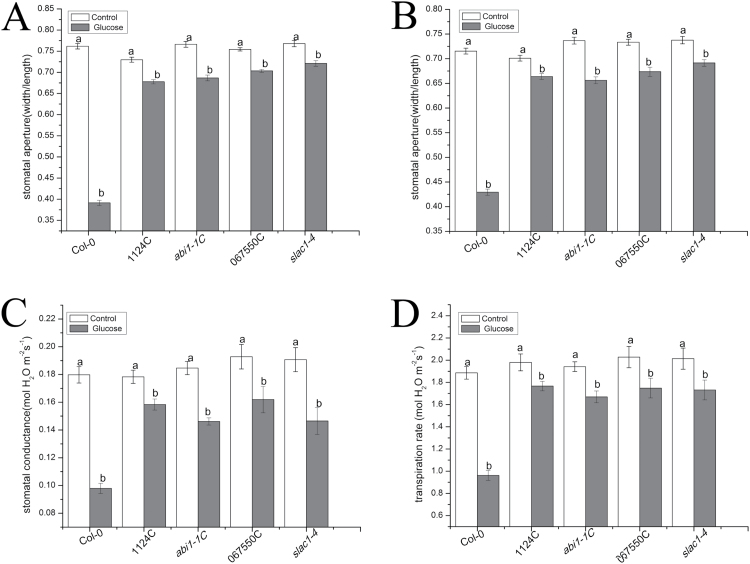
Glucose-induced stomatal closure was mediated by functional PYR/RCAR, ABI1, OST1, and SLAC1 in Arabidopsis. (A) Effects of glucose on stomatal apertures in epidermal strips of wild-type Arabidopsis and ABA signaling mutants. Abaxial epidermal strips of Arabidopsis were pre-incubated in MES–KCl buffer for 2 h under light and then were separately floated on 100 mM glucose. Stomatal apertures were measured 2 h later. Each bar demonstrates the means ±SE of three biological repeats (*n*=150). (B) Effects of glucose on stomatal apertures in intact leaves of wild-type Arabidopsis and ABA signaling mutants. The intact leaves of wild-type Arabidopsis and its mutants kept under light for 3 h were treated with 100 mM glucose and sterile water; after 2 h, the abaxial epidermal strips were peeled off and immediately observed under a microscope. Each bar indicates the means ±SE from three independent experiments (*n*=150). (C and D) Alterations in leaf *g*_s_ and E of wild-type Arabidopsis and ABA signaling mutants in response to sterile water and 100 mM glucose. Each bar indicates the means ±SE (*n*=6). Different letters above the bars denote statistically significant differences among treatments (LSD test, *P*<0.05). White bar, control; gray bar, glucose.

We used several mutations in the Col-0 background in the ABA signaling pathway through PYR/RCAR receptors, namely *pyr1pyl1pyl2pyl4* (1124C), *abi1-1* (Salk_076309C), *ost1* (Salk_0677550C), and *slac1-4* (Salk_137265), to explore whether ABA signaling components through PYR/RCAR receptors are involved in regulating stomatal responses to glucose. As is shown in Fig. 4A, the glucose-induced stomatal closure in epidermal peels was greatly inhibited in 1124C (*P*<0.001), Salk_076309C (*P*<0.001), Salk_0677550C (*P*<0.001), and *slac1-4* (*P*<0.001) mutants in the Col-0 background. Furthermore, 100 mM glucose also induced stomatal closure in intact leaves of Col-0 (*P*<0.001) (Fig. 4B). However, the stomatal closure was significantly impaired in 1124C (*P*<0.001), Salk_076309C (*P*<0.001), Salk_067550C (*P*<0.001), and *slac1-4* (*P*<0.001) mutants (Fig. 4B). Leaf *g*_s_ and E under steady-state conditions in various ABA signalosome mutants were monitored with a gas exchange system to survey the roles of ABA signalosomes through PYR/RCAR receptors in glucose-induced stomatal closure in Arabidopsis. Compared with the control treatment, leaf *g*_s_ of Col-0, 1124C, Salk_076309C, Salk_067550C, and *slac1-4* decreased by 45.5% (*P*<0.001), 11.2% (*P*=0.064), 19.6% (*P*=0.0030), and 23.1% (*P*<0.001), respectively, in response to 100 mM glucose application (Fig. 4C). Correspondingly, the leaf E was severally reduced by 49.0% (*P*<0.001), 16.9% (*P*<0.001), 10.8% (*P*=0.014), and 14.0% (*P*=0.021) (Fig. 4D). Considering all the results, we speculated that ABA signaling components such as PYR/RCAR, ABI1, OST1, and SLAC1 were required for glucose-triggered stomatal closure.

### The effects of CAT, DPI, and SHAM on glucose-induced stomatal closure and ROS production in Arabidopsis

**Fig. 5.**
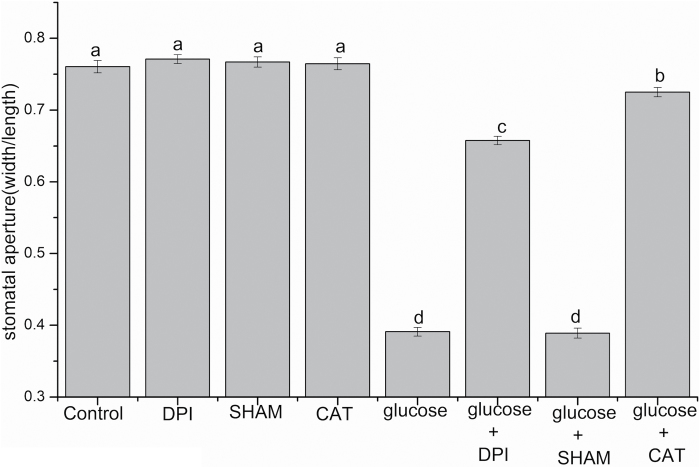
Effects of 20 µM DPI, 2 mM SHAM, and 100 U ml^−1^ CAT on glucose-induced stomatal closure in Arabidopsis. Abaxial epidermal strips of Arabidopsis were pre-incubated in opening buffer for 2 h under light, and then were transferred to MES–KCl buffer in the presence or absence of 20 µM DPI, 2 mM SHAM, and 100 U ml^−1^ CAT, and remained there for 30 min. They were then immersed in 100 mM glucose solution. Stomatal apertures were measured 2 h later. These data are the mean ±SE of three biological repeats (*n*=150 per bar). Different letters above the bars demonstrate mean values that are significantly different from one another, according to ANOVA (LSD test, *P*<0.05).

**Fig. 6.**
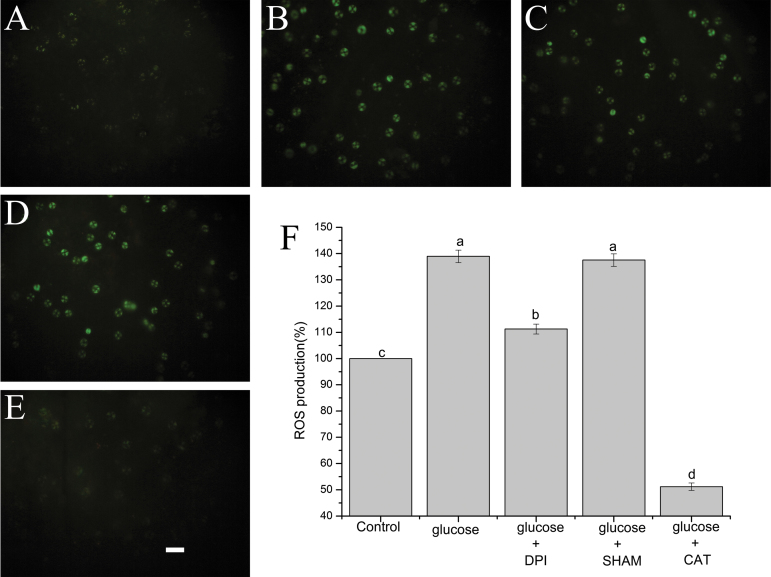
Glucose triggered ROS production in Arabidopsis. Abaxial epidermal strips of Arabidopsis were treated with glucose alone, or glucose in the presence of DPI, SHAM, and CAT for 120 min. They were then loaded with 50 μM H_2_DCF-DA in the dark. After 15–20 min, the strips were washed three times with MES–KCl buffer, and representative pairs of guard cells were photographed using fluorescence microscopy (A–E). The scale bar in (E) is 40 μm, which applies to all photographs. (F) Analysis of fluorescence intensities of guard cells in images (A–E). The vertical scale indicates the percentage of H_2_DCF-DA fluorescent levels, taking 100% as the value of control treatments. These data are the mean ±SE from three independent experiments (*n*=100). Different letters above the bars represent mean values that are statistically different from one another as determined by ANOVA (LSD test, *P*<0.05).

To test whether ROS are involved in glucose-induced stomatal closure in Arabidopsis and which enzyme catalyzes ROS production, we assessed the effects of DPI, SHAM, and CAT on glucose-induced stomatal closure and ROS production. As is shown in Fig. 5, in contrast to the treatment with glucose alone, the glucose-induced stomatal closure was almost completely inhibited by a ROS scavenger, CAT, at 100 U ml^−1^ (*P*<0.001). Additionally, it was greatly reversed by an NADPH oxidase inhibitor, DPI, at 20 µM (*P*<0.001), while it was not suppressed by a peroxidase inhibitor, SHAM, at 2 mM (*P*=0.828) (Fig. 5). However, applying DPI, SHAM, or CAT alone caused no statistically significant alterations in stomatal aperture (Fig. 5). Furthermore, we tested the glucose-induced ROS production in guard cells of Arabidopsis by loading H_2_DCF-DA. Figure 6 shows that application of 100 mM glucose solution significantly improved ROS production compared with the control (*P*<0.001). ROS were almost entirely removed by 100 U ml^–1^ CAT (*P*<0.001), and significantly abolished by 20 µM DPI (*P*<0.001), while they were not affected by 2 mM SHAM (*P*=0.584) (Fig. 6). These results were in accordance with the stomatal response shown in Fig. 5, suggesting that glucose-triggered stomatal closure was mediated by ROS production mostly via DPI-sensitive NADPH oxidase but not SHAM-sensitive peroxidase in Arabidopsis. These phenomena are similar to our previous findings in *V. faba* that *Chlorella*- and glucose-elicited stomatal closure relies on ROS production mainly mediated by NADPH oxidase (Li *et al.*, 2014, 2016).

### [Ca^2+^]_cyt_- and NR-mediated NO production are responsible for glucose-induced stomatal closure in Arabidopsis

**Fig. 7.**
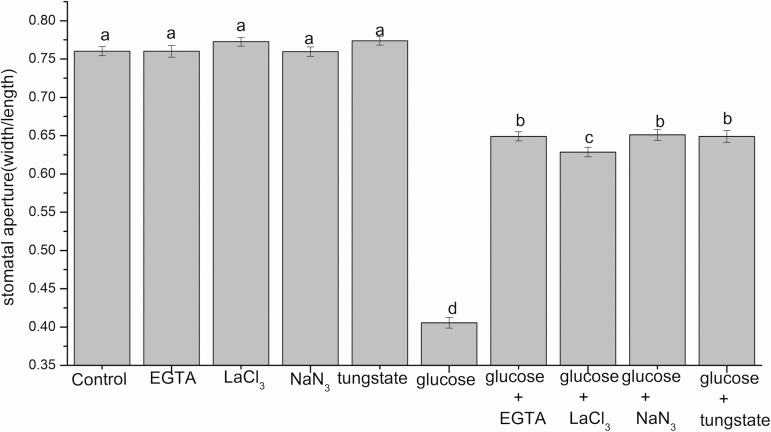
The effects of EGTA, LaCl_3_, tungstate, and NaN_3_ on glucose-triggered stomatal closure. Abaxial epidermal strips of Arabidopsis were pre-incubated in MES–KCl buffer for 2 h under light, and then were treated with 2 mM EGTA, 1 mM LaCl_3_, 100 µM tungstate, and 2 mM NaN_3_ for 30 min. They were then floated on 100 mM glucose for 2 h, and the stomatal apertures were assessed after 2 h. Each bar demonstrates the means ±SE of three biological repeats (*n*=150). Different letters above the bars indicate statistically significant significances among treatments as determined by ANOVA (LSD test, *P*<0.05).

**Fig. 8.**
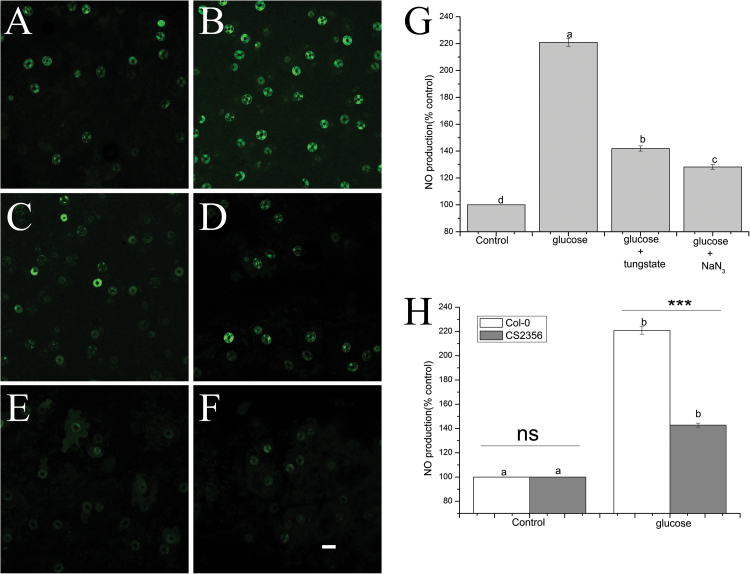
Glucose induced NO production in wild-type Arabidopsis Col-0 and *nia1-1nia2-5* mutants. Abaxial epidermal strips of Arabidopsis were treated with glucose alone, or glucose in the presence of tungstate and NaN_3_ for 120 min. They were then loaded with 10 μM DAF-FM DA in the dark for 30 min. After a brief wash with MES–KCl buffer, the strips were observed, and representative pairs of guard cells were photographed using a confocal laser scanning microscopy (A–F). The scale bar in (F) is 20 μm, which applies to all photographs. (G and H) Quantification assay of DAF-FM DA fluorescence intensities of guard cells in images (A–F). The vertical scale represents the percentage of DAF-FM DA fluorescent levels, taking 100% as the value of control treatments. These data represent the mean ±SE of three biological replicates (*n*=60). Different letters above the bars represent mean values that are statistically significantly different from one another as determined by ANOVA (LSD test, *P*<0.05). ns, non-signiﬁcant differences; ***, signiﬁcant differences at *P*<0.001.

We assessed the effects of EGTA (a Ca^2+^ chelator) and LaCl_3_ (a Ca^2+^ channel blocker) on glucose-induced stomatal closure to clarify whether glucose-triggered stomatal closure depends on [Ca^2+^]_cyt_ and a Ca^2+^ channel in Arabidopsis. As is shown in Fig. 7, the glucose-induced stomatal closure was greatly impaired by 2 mM EGTA (*P*<0.001) and 1 mM LaCl_3_ (*P*<0.001). However, the stomatal aperture showed no statistically significant differences when EGTA and LaCl_3_ were used alone (Fig. 7). These results reveal that [Ca^2+^]_cyt_ and the Ca^2+^ channel may be involved in glucose-induced stomatal closure in Arabidopsis. The effects of two NR inhibitors, tungstate and NaN_3_, on glucose-induced stomatal closure were examined to determine whether NO is necessary for glucose-triggered stomatal closure in Arabidopsis and what the enzyme source of NO production is. Figure 7 shows that the glucose-triggered stomatal closure was significantly inhibited by 100 µM tungstate (*P*<0.001) and 2 mM NaN_3_ (*P*<0.001). However, treatment with tungstate or NaN_3_ alone caused no statistically significant differences in stomatal aperture (Fig. 7). Furthermore, we monitored the glucose-induced NO production in guard cells of Arabidopsis. It was observed that with 100 mM glucose treatment, NO production was greatly increased compared with the control (*P*<0.001) (Fig. 8). However, the NO accumulation was largely removed by tungstate (*P*<0.001) and NaN_3_ (*P*<0.001) (Fig. 8). These results were consistent with the stomatal response noted in Fig. 7. In contrast, the NO production induced by glucose was greatly inhibited in NR-null mutant *nia1-1nia2-5* plants (Fig. 8), indicating that glucose-induced stomatal closure was dependent on NO production mainly mediated by NR in Arabidopsis.

### Glucose-induced stomatal closure is mediated by functional CDPK6 and NR

**Fig. 9.**
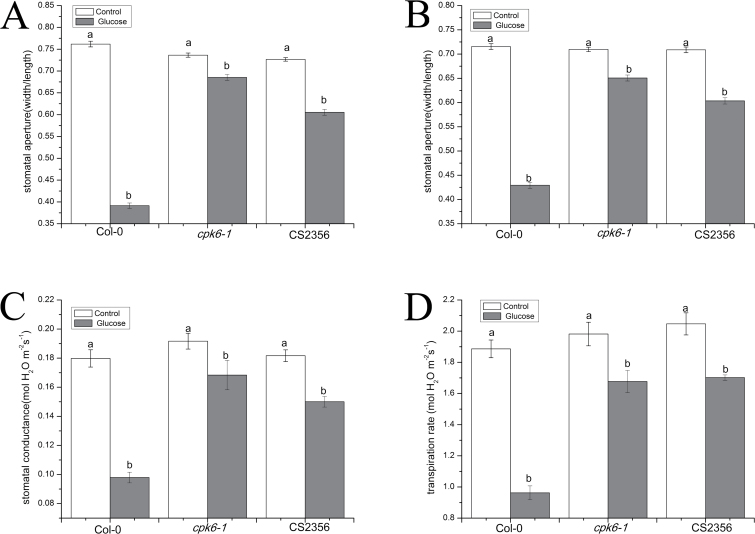
Glucose-induced stomatal closure was dependent on functional CDPK6 and NR in Arabidopsis. (A) Glucose-induced stomatal closure in epidermal strips of wild-type Arabidopsis Col-0, *cpk6*, and *nia1nia2* mutants. Abaxial epidermal strips of Arabidopsis were pre-incubated in MES–KCl buffer under light for 2 h, and were immersed in 100 mM glucose solution for another 2 h. The stomatal apertures were then tested. These data indicate the means ±SE from three independent experiments (*n*=150 per bar). (B) Glucose-triggered stomatal closure in whole leaves of Col-0, *cpk6*, and *nia1nia2* mutants. Intact leaves of wild-type Arabidopsis and its mutants acclimated to light for 3 h were treated with sterile water and 100 mM glucose solution. Two hours later, the abaxial epidermal strips were promptly peeled off and observed under a microscope. Each bar indicates the means ±SE from three independent experiments (*n*=150). (C and D) Changes in leaf *g*_s_ and E of wild-type Arabidopsis and its mutants in response to sterile water and 100 mM glucose. These data represent the mean ±SE (*n*=6 per bar). Different letters above the bars represent significant differences among treatments as determined by ANOVA (LSD test, *P*<0.05). White bar, control; gray bar, glucose.

The effect of glucose on stomatal aperture of two Arabidopsis mutants, *cpk6-1* and *nia1-1nia2-5*, in the Col-0 background was determined to confirm the influence of CPDK6 and NR on glucose-induced stomatal closure. It was observed that application of 100 mM glucose caused the stomatal apertures to decrease by 48.6% (*P*<0.001), 15.3% (*P*<0.001), and 20.9% (*P*<0.001) in epidermal peels of Col-0, *cpk6-1*, and *nia1-1nia2-5* plants , respectively, after 2 h (Fig. 9A), in contrast to the control treatment. The stomatal apertures were individually reduced by 40.0% (*P*<0.001), 16.7% (*P*<0.001), and 19.8% (*P*<0.001) when leaves of Col-0, *cpk6-1*, and *nia1-1nia2-5* plants were treated with 100 mM glucose for 2 h (Fig. 9B). Leaf *g*_s_ and E of Col-0, *cpk6-1*, and *nia1-1nia2-5* plants under steady-state conditions were further measured to assess the contribution of CDPK6 and NR to the regulation of stomatal movement in response to glucose treatments in Arabidopsis. It was shown that leaf *g*_s_ was decreased by 49.2% (*P*<0.001), 12.2% (*P*=0.033), and 17.4% (*P*<0.001) when 100 mM glucose was applied to intact rosettes of Col-0, *cpk6-1*, and *nia1-1nia2-5* plants, respectively, followed by 55.4% (*P*<0.001), 15.4% (*P*=0.0055), and 16.9% (*P*<0.001) reductions in E (Fig. 9C, D), implying that CDPK6 and NR may participate in the regulation of stomatal movement by glucose.

## Discussion

### Glucose-induced stomatal closure in Arabidopsis

Previous studies have shown that ABA can induce stomatal closure and suppress stomatal opening by regulating a series of complex signaling pathways in guard cells (Merlot *et al.*, 2001; Underwood *et al.*, 2007; Acharya and Assmann, 2009). It has been further demonstrated that there are some unexpected overlaps between ABA and glucose signaling pathways (Rolland *et al.*, 2002, 2006; Zhang *et al.*, 2008). Interestingly, our present work showed that glucose triggered stomatal closure in Arabidopsis (Fig. 1), which agreed with a previous phenomenon found in tomato (Kelly *et al.*, 2013) and our recent findings in *V. faba* (Li *et al.*, 2016). Nevertheless, our findings were contrary to the previous theories that glucose or other carbohydrates were ineffective in inhibiting stomatal opening, which was concluded to be the case in *Tulipa gesneriana* L. and *V. faba* L. (Dittrich and Mayer, 1978). It is well known that stomatal closure and suppression of stomatal opening in response to external stimuli are two separate processes regulated by different signaling transduction pathways (Allen *et al.*, 1999; Wang *et al.*, 2001; Mishra *et al.*, 2006), which may provide clarification of the inconsistency of the data. Figure 1 also showed dosage and time effects of glucose-induced stomatal closure, and the maximum effect appeared with 100 mM glucose and 2 h treatment. Our published data indicated that glucose induced stomatal closure in a dose- and time-dependent manner in *V. faba* (Li *et al.*, 2016). Compared with the dosage effects of glucose on stomatal aperture between *V. faba* and Arabidopsis, applications of 25, 50, and 100 mM glucose induced corresponding reductions in stomatal aperture. However, there were significant differences in the reductions of stomatal apertures after treatment with 1, 150, and 200 mM glucose compared with those results from *V. faba* and Arabidopsis. This may be attributed to different stomatal anatomy and responses between Arabidopsis and *V. faba*. These results were consistent with some studies showing that some stimuli such as ABA, salicylic acid (SA), hydrogen peroxide (H_2_O_2_), ethylene, and UV-B can induce stomatal closure in both *V. faba* and Arabidopsis (Zhang *et al.*, 2001; Bright *et al.*, 2006; Desikan *et al.*, 2006; Mori *et al.*, 2006; He *et al.*, 2011, 2013). Furthermore, Fig. 1 showed that the effects of glucose on stomatal closure were less significant at higher concentrations, and 200 mM mannitol had no obvious influence on stomatal aperture. This phenomenon implied that glucose-triggered stomatal closure was not due to osmotic stress caused by glucose but rather to signaling events. It was similar to the recent findings that sucrose stimulated stomatal closure mediated by HXK and ABA in an osmotic stress-independent manner in tomato and Arabidopsis (Kelly *et al.*, 2013). To illustrate further the mechanisms underlying glucose-triggered stomatal closure, more experiments will be carried out using pharmacological methods and the stomatal system of Arabidopsis mutants.

### Functional GIN1 and GIN2 are involved in glucose-induced stomatal closure

Recent studies have revealed that HXK1 plays significant roles in mediating stomatal closure in response to sucrose in Arabidopsis, tomato, and citrus plants (Kelly *et al.*, 2013; Lugassi *et al.*, 2015). The *gin2-1* mutant is a nonsense mutation which has reduced HXK1 catalytic activity (Moore *et al.*, 2003). The mutant is specific to glucose insensitivity but not to osmotic changes. Phenotype analysis revealed that high-glucose repression of cotyledon expansion, chlorophyll accumulation, true-leaf development, and root elongation were impaired in the *gin2-1* mutant (Moore *et al.*, 2003). Moreover, previous studies have indicated that the *gin2-1* mutant has a higher *g*_s_ and E; however, AtHXK1-overexpressing plants had reduced *g*_s_ and E compared with the wild type (Kelly *et al.*, 2013). Our present work showed similar results in that the *gin2-1* mutant displayed higher *g*_s_ and E compared with the wild type (Fig. 2). Additionally, Fig. 2 also shows that the *g*_s_ and E of the *gin2-1* mutant had no obvious changes in response to glucose treatment, and glucose-induced stomatal closure was greatly restrained in epidermal peels and intact leaves of *gin2-1*. These results indicated that glucose-triggered stomatal closure was dependent on GIN2, as was the case for sucrose (Kelly *et al.*, 2013; Lugassi *et al.*, 2015). In addition, genetic analysis revealed that GIN1 acted downstream of the glucose sensor HXK1 and gin1 was epistatic to HXK1 in the glucose signaling pathway (Zhou *et al.*, 1998). Phenotype analysis indicated that the *gin1-1* mutant was insensitive to glucose and had a greater rate of water loss, which induced the symptoms of wilting and withering, especially under low relative humidity and water stress (W.-H. Cheng *et al.*, 2002). In our present work, we observed that the *gin1-1* mutant had higher stomatal aperture, *g*_s_, and E (Fig. 3). This may provide rational explanations for the findings by W.-H. Cheng *et al.* (2002). Glucose-induced reduction in the stomatal aperture, *g*_s_, and E was almost completely abolished in the *gin1-1* mutant (Fig. 3), suggesting that GIN1 may be required in glucose-induced stomatal closure.

### Glucose-induced stomatal closure is modulated by functional PYR/RCAR receptors, ABI1, OST1, and SLAC1

ABA plays vital roles in mediating stomatal closure in response to various environmental stimuli (Ma *et al.*, 2009). The mechanisms underlying ABA perception by the ABA receptors PYL/RCAR and its downstream signaling network have been identified recently (Ma *et al.*, 2009; Park *et al.*, 2009). However, whether PYR/RCAR receptors and downstream signaling components, such as PP2C, OST1, and SLAC1, are required for stomatal closure induced by glucose treatments remains unknown. The present work showed that glucose-triggered stomatal closure was greatly impaired in epidermal peels and intact leaves of *pyr1pyl1pyl2pyl4*, *abi1*, *ost1*, and *slac1-4* mutants (Fig. 4A, B). Furthermore, reductions in *g*_s_ and E caused by glucose were significantly inhibited in the above mutants (Fig. 4C, D). These results suggest that glucose-mediated stomatal responses may be dependent on ABA signaling components through PYR/RCAR receptors. It is similar to the signaling pathway where CO_2_, O_3_, Ca^2+^, H_2_O_2_, and NO induce stomatal closure (Negi *et al.*, 2008; Vahisalu *et al.*, 2008). The partial inhibition of glucose-triggered stomatal closure may be ascribed to genetic redundancies among PYR/RCAR, PP2C, OST1, and SLAC1 proteins (Szostkiewicz *et al.*, 2010). Furthermore, OST1 is observed to be active in response to some stimuli independent of ABA and PYR/RCAR receptors, which explains the partial stomatal responses to glucose treatments in the *ost1* mutant (Xie *et al.*, 2006; Yoshida *et al.*, 2006; Boudsocq *et al.*, 2007). Recently, SLAC1 was demonstrated to be activated by CDPKs in addition to OST1 (Ye *et al.*, 2013), which further explains the partial impairment of stomatal closure induced by glucose in the *ost1* mutant. In addition to SLAC1, other anion channels such as the voltage-dependent rapid-type anion channel QUAC1 and the slow-type anion channel have been reported to be involved in stomatal closure (Meyer *et al.*, 2010; Imes *et al.*, 2013). This phenomenon provided an alternative explanation for the partial inhibition of glucose-induced stomatal closure in the *slac1-4* mutant.

### Glucose-induced stomatal closure is mediated by ROS production in guard cells of Arabidopsis

ROS have been well established as vital second messengers in regulating stomatal closure in response to diverse stimuli (Mustilli *et al.*, 2002; Munemasa *et al.*, 2007). In the present study, we found that optimal concentrations of glucose could noticeably increase the level of ROS in guard cells of Arabidopsis (Fig. 6B), just as ABA and MeJA did (Kwak *et al.*, 2003; Desikan *et al.*, 2004; Suhita *et al.*, 2004). Pharmacological experiments indicated that glucose-induced ROS production was almost totally removed by a membrane-impermeable ROS scavenger, CAT, in Arabidopsis (Fig. 6E). These results imply that glucose-induced ROS production may function outside the plasma membrane of guard cells, due to the high permeability of the plasma membrane to H_2_O_2_ (Lee *et al.*, 1999; Zhang *et al.*, 2001; Munemasa *et al.*, 2007). In plants, ROS can be induced via different enzymes in response to various stimuli (Pei *et al.*, 2000; Munemasa *et al.*, 2007). These enzymes include NADPH oxidases, cell wall-localized peroxidases, xanthine oxidases, oxalate oxidases, and amine oxidases (Luis *et al.*, 2002; Vranová *et al.*, 2002; Kwak *et al.*, 2003; Cuevas *et al.*, 2004; Cona *et al.*, 2006). Among these enzymes, NADPH oxidase and peroxidase have been the most studied. For instance, NADPH oxidases have been shown to mediate stomatal closure induced by ABA, MeJA, ozone, darkness, ethylene, allyl isothiocyanate, a low dose of UV-B, flg22, lipopolysaccharide (LPS), elf18, and *Chlorella* (Kwak *et al.*, 2003; Desikan *et al.*, 2004; Suhita *et al.*, 2004; Joo *et al.*, 2005; Bright *et al.*, 2006; Melotto *et al.*, 2006; Munemasa *et al.*, 2007; Khokon *et al.*, 2011; Sawinski *et al.*, 2013; Li *et al.*, 2014). Peroxidases are proved to modulate stomatal closure triggered by SA, a high dose of UV-B, chitosan, yeast elicitor (YEL), methylglyoxal, and yeast (Mori *et al.*, 2001; Khokon *et al.*, 2010*b*; He *et al.*, 2011; Hoque *et al.*, 2012). The present work indicated that glucose-induced ROS production was greatly suppressed by an NADPH oxidase inhibitor, DPI, while it was not affected by a peroxidase inhibitor, SHAM (Fig. 6). These results suggest that ROS production induced by glucose is mainly mediated by NADPH oxidases in Arabidopsis. It is similar to the phenomena observed in cultured vascular cells (Inoguchi *et al.*, 2000, 2003). In addition, our results showed that glucose-triggered stomatal closure was almost completely restored by CAT, strongly inhibited by DPI, and not impaired by SHAM (Fig. 5). It coincided with the effects of CAT, DPI, and SHAM on ROS production stimulated by glucose, which was shown in Fig. 6. These results indicate that glucose-triggered stomatal closure is mainly mediated by ROS production via DPI-sensitive plasma membrane NADPH oxidases but not SHAM-sensitive peroxidases in Arabidopsis. This mechanism is consistent with our recent findings in *V. faba* (Li *et al.*, 2016). Previous studies have revealed that OST1 kinase can phosphorylate the plasma membrane NADPH oxidase (Sirichandra *et al.*, 2009). As is shown in Fig. 4, reductions in stomatal aperture, *g*_s_, and E caused by glucose were restored in the *ost1* mutant, implying that OST1 is involved in the glucose-triggered stomatal closure. Based on the previous and present results, we speculate that glucose-induced ROS production and stomatal closure are dependent on OST1-activated NADPH oxidase.

### Glucose-induced stomatal closure is mediated by [Ca^2+^]_cyt_ and CDPK6

Previous studies have documented that [Ca^2+^]_cyt_ functions in ABA and glucose signaling transduction that controls plant growth, development, and stress responses (S.H. Cheng *et al.*, 2002). [Ca^2+^]_cyt_ is a vital secondary messenger in ABA-dependent stomatal closure (Mori *et al.*, 2006). In the present work, we found that glucose-induced stomatal closure was significantly repressed by LaCl_3_ (a Ca^2+^ channel blocker) and EGTA (a Ca^2+^ chelator) (Fig. 7). These results reveal that glucose-induced stomatal closure is dependent on [Ca^2+^]_cyt_ in Arabidopsis, which is similar to our findings in *V. faba* (Li *et al.*, 2016). This pathway is the same as that by which ABA triggers stomatal closure mediated by [Ca^2+^]_cyt_ (Srivastava *et al.*, 2009; Ye *et al.*, 2013). CDPKs are confirmed to play vital roles in the Ca^2+^-dependent signaling pathway (Mori *et al.*, 2006; Zhu *et al.*, 2007; Geiger *et al.*, 2009; Brandt *et al.*, 2012). In guard cells, CDPKs mediate stomatal closure mainly via the activation of S-type and Ca^2+^ channels, as well as via the inhibition of inwardly rectifying potassium (K_in_) channels (Mori *et al.*, 2006; Zou *et al.*, 2010; Munemasa *et al.*, 2011). For instance, CDPK3 and CDPK6 can activate Ca^2+^-permeable channels and S-type channels in response to ABA and Ca^2+^, inducing stomatal closure (Mori *et al.*, 2006). In our present work, we observed that glucose-triggered stomatal closure was impaired in epidermal peels and intact leaves of the *cpk6-1* mutant (Fig. 9A, B). In addition, the reductions in *g*_s_ and E were restored in the *cpk6-1* mutant in response to glucose (Fig. 9C, D). These findings imply that CDPK6 is required for glucose-induced stomatal closure, consistent with previous findings that CDPK6 participates in Ca^2+^-, ABA-, MeJA-, and YEL-triggered stomatal closure (Mori *et al.*, 2006; Munemasa *et al.*, 2011; Ye *et al.*, 2013). Nevertheless, the partial impairments of stomatal responses to glucose in the *cpk6-1* mutant are likely to be ascribed to genetic redundancies of CDPKs. An alternative explanation is that a Ca^2+^-independent parallel signaling pathway may be involved in glucose-induced stomatal closure.

### Glucose-induced stomatal closure is dependent on NO and NR

NO has been recognized as a key secondary messenger in ABA- and microbe-associated molecular pattern (MAMP)-mediated stomatal closure and functions downstream of ROS production (Neill *et al.*, 2002; Garcia-Mata and Lamattina, 2007; Khokon *et al.*, 2010*b*). It has also been reported that sucrose can elicit stomatal closure by stimulating guard cell-located NO production via HXK (Kelly *et al.*, 2013). However, the source of NO in plants and the role of NO in stomatal closure are still controversial (Lozano-Juste and León, 2010; Yu *et al.*, 2012). Notably, our studies showed that glucose induced NO production in guard cells of Arabidopsis (Fig. 8). This result was accompanied by stomatal closure (Fig. 7), as also caused by yeast, LPS, and sucrose (Melotto *et al.*, 2006; Gao *et al.*, 2013; Kelly *et al.*, 2013; Sawinski *et al.*, 2013). In addition, glucose-induced NO production was greatly reduced by two NR inhibitors, tungstate and NaN_3_, followed by reversal of the corresponding stomatal closure (Figs 7, 8). These pharmacological data suggest that glucose-induced NO production and stomatal closure are mainly mediated by NR. Moreover, glucose-induced NO accumulation and stomatal closure were significantly abolished in NR-null mutant *nia1-1nia2-5* plants (Fig. 8). Unlike the wild type, the *nia1-1nia2-5* mutant greatly reversed the reductions in stomatal aperture, *g*_s_, and E in response to glucose (Fig. 9). Genetic evidence further reveals that NR is the main source of NO production responsible for glucose-triggered stomatal closure. Recently, it has been revealed that ABA-triggered stomatal closure and inhibition of opening are not affected in the NO-deficient mutant *nia1 nia2 noa1-2* (Lozano-Juste and León, 2010). As is noted in Fig. 9, the stomatal responses to glucose were partially inhibited in the *nia1-1nia2-5* mutant, implying that NR-independent NO or an NO-independent pathway might be involved in glucose-triggered stomatal closure. Whether a nitric oxide synthase (NOS)-like enzyme is another source of NO required for glucose-induced stomatal closure remains an open question and needs to be answered in the future.

In conclusion, glucose could induce stomatal closure, showing dosage and time effects in epidermal strips of Arabidopsis. ROS production via NADPH oxidases, [Ca^2+^]_cyt_, a Ca^2+^ channel, and NR-mediated NO production were responsible for glucose-induced stomatal closure in Arabidopsis. Glucose-induced stomatal closure was mediated by GIN1, GIN2, PYR/RCAR, PP2C, OST1, CDPK6, NR, and SLAC1 in Arabidopsis. This study may provide new evidence for the involvement of ABA and sugar signaling in glucose-triggered stomatal closure.

## Author contributions

YL and GW conceived and designed the experiments; YL, SX, ZW, LH, KX, and GW performed the experiments; YL, SX, and GW analyzed the data; YL, ZW, LH, KX, SX, and GW contributed reagents/materials/analysis tools; and YL, SX, and GW wrote the paper.

## Abbreviations:

ABA: abscisic acid
CAT: catalase
[Ca^2+^]_cyt_: cytosolic free calcium concentration
CDPK6: Ca^2+^-dependent protein kinase 6
DAF-FM DA: 4-amino-5-methylamino-2',7'-difluorofluorescein diacetate
DPI: diphenylene iodonium chloride
GIN1: glucose insensitive 1
GIN2: glucose insensitive 2
H_2_DCF-DA: 2', 7'-dichlorofluorescein diacetate
H_2_O_2_: hydrogen peroxide
LaCl_3_: lanthanum chloride
NaN_3_: sodium azide
NO: nitric oxide
NR: nitrate reductase
OST1: open stomata 1
ROS: reactive oxygen species
SHAM: salicylhydroxamic acid
SLAC1: slow type anion channel 1.
